# Social, Emotional, and Behavioral Skills: Age and Gender Differences at 12 to 19 Years Old

**DOI:** 10.3390/jintelligence11060118

**Published:** 2023-06-13

**Authors:** Tommaso Feraco, Chiara Meneghetti

**Affiliations:** Department of General Psychology, University of Padova, 35122 Padova, Italy

**Keywords:** 21st century skills, soft skills, SEB skills, socioemotional competencies, noncognitive skills, BESSI, sex differences, adolescence, gender, development

## Abstract

Individuals use social, emotional, and behavioral (SEB) skills to build and maintain social relationships, regulate emotions, and manage goal-directed behaviors. A promising integrative framework of SEB skills was recently proposed, showing that they matter for positive outcomes during adolescence. Nothing is known about how and whether they differ between 12 and 19 years old and whether such differences depend on gender (males or females). Uncovering their age trajectories is fundamental because SEB skills are highly needed during this period of life. Educators, psychologists, and policymakers need to understand when, why, and how interventions concerning SEB skills should be proposed, potentially considering male and female profiles. To cover this gap, we cross-sectionally analyzed data from 4106 participants (2215 females, 12–19 years old). We highlighted age and gender differences in the five domains of SEB skills (self-management, innovation, cooperation, social engagement, and emotional resilience). Our results show that each SEB skill follows a specific age trend: emotional resilience and cooperation skills increase naturally between 12 and 19 years old, while innovation, social engagement, and self-management skills decline, especially between 12 and 16 years old, and grow later. The trajectories of self-management, social engagement, and emotional resilience skills also differ between males and females. Importantly, we detected declines in SEB skills (especially for social engagement and innovation skills) that can inform policies and interventions to sustain SEB skills in youths to favor their well-being and success in this crucial period.

## 1. Introduction

Psychological constructs are not fixed. They tend to change across the lifespan, especially during childhood and adolescence (Sawyer et al. 2018; Yurgelun-Todd 2007). This is true for cognitive abilities, personality traits, character strengths, and self-esteem (Costa et al. 2019; Gur et al. 2012; Soto 2016). However, patterns of development/change are different between constructs, facets of the same construct, and even between populations. For example, males and females often show small differences in the levels/scores of specific traits or abilities and in the trajectory that such scores follow across the lifespan (Feraco and Cona 2022; Gur et al. 2012; Hills and Byrne 2010; Miller and Halpern 2014; Roberts and Yoon 2022; Soto and Tackett 2015). Therefore, considering the variation in psychological characteristics between populations, such as males and females, is essential.

For the first time, in this study, we will focus on the cross-sectional trajectories of social, emotional, and behavioral (SEB) skills, as measured according to the new emerging theoretical framework proposed by Soto et al. (2022a), in a large sample of 4106 male and female adolescents (12 to 19 years old). This is crucial because adolescence is hypothesized to involve high use and malleability of SEB skills (Napolitano et al. 2021; Soto et al. 2021), whose importance has been recently documented (Soto et al. 2022b). Understanding whether and how they change might inform teachers, policymakers, psychologists, and educators about which skills are suitable for improvement or need significant attention, also distinguishing between males and females, who are known to face different situations and challenges (under biological, social, and developmental aspects) during this crucial period of life (Bramen et al. 2011; Dahl 2004; Sawyer et al. 2018).

### 1.1. Social, Emotional, and Behavioral Skills

SEB skills have recently been defined as people’s capacity to regulate emotions, learn from experience, manage goal-directed behaviors, and build and maintain good social relationships (Soto et al. 2021, 2022a). Differently from other constructs such as personality traits, character strengths, or CASEL competencies, SEB skills do not represent how people *tend* to think, feel, and behave in general but specifically focus on the individual’s *capability* to think, feel, and act in a certain way when the situation calls for it (Soto et al. 2021). In other words, it is not whether I usually behave in a certain way but whether I can consciously activate a behavior when needed. For example, I might be generally shy and introverted, but I can easily start talking and interacting efficiently with other people when the situation requires it. Despite this theoretical difference, capabilities (i.e., skills) and tendencies (i.e., personality traits) are related because a person who tends to act in a certain way will also acquire the corresponding ability more easily (Soto et al. 2021). SEB skills, however, showed divergent validity from all these constructs and incremental validity beyond them. Specifically, in the first studies on the topic, SEB skills were incrementally important during adolescence for a host of positive outcomes, such as academic achievement, school engagement, social relationships, and well-being (Lechner et al. 2022; Soto et al. 2022a, 2022b). These results highlight the importance of studying these skills, particularly because SEB skills can be learned and developed, thus providing room for interventions.

#### 1.1.1. The Framework and Measurement of SEB Skill

Despite the importance that SEB skills—frequently called “soft skills,” “character skills,” or “non-cognitive skills”—have for policymaking, research, and practice (European Commission 2016; Feraco et al. 2023b; Robles 2012; World Economic Forum 2016), an integrative psychological framework and a clear measurement method of these skills were missing from the literature. Soto and colleagues (Soto et al. 2022a, 2022b) recently faced this issue and proposed a framework that consolidates and integrates previous conceptualizations of SEB skills. They developed the Behavioral, Emotional, and Social Skills Inventory (BESSI), which offers “what is arguably the most comprehensive and fine-grained framework for assessing SEB skills to date” (Lechner et al. 2022, p. 2). The framework encompasses 32 skill facets (e.g., leadership skill, creative skill, capacity for trust) that are grouped into five higher-order domains: self-management skills (SMD), innovation skills (IND), cooperation skills (COD), social engagement skills (SED), and emotional resilience skills (ESD).

- Self-management skills (e.g., task management, time management, goal regulation) encompass the skills people use to effectively set, plan, and reach their goals or complete tasks.
- Innovation skills (e.g., abstract thinking and creative skills) are people’s capacities to handle, learn, create, and engage with new ideas and experiences.
- Cooperation skills (e.g., teamwork and perspective-taking) are the abilities people use to build and maintain positive social relationships.
- Social engagement skills (e.g., leadership and conversation) are the abilities that people use to communicate and actively engage with others.
- Emotional resilience skills (e.g., stress regulation and impulse regulation skills) are skills that people use to regulate their emotions and moods efficiently, depending on the requests and situations at hand.

**Figure 1:**
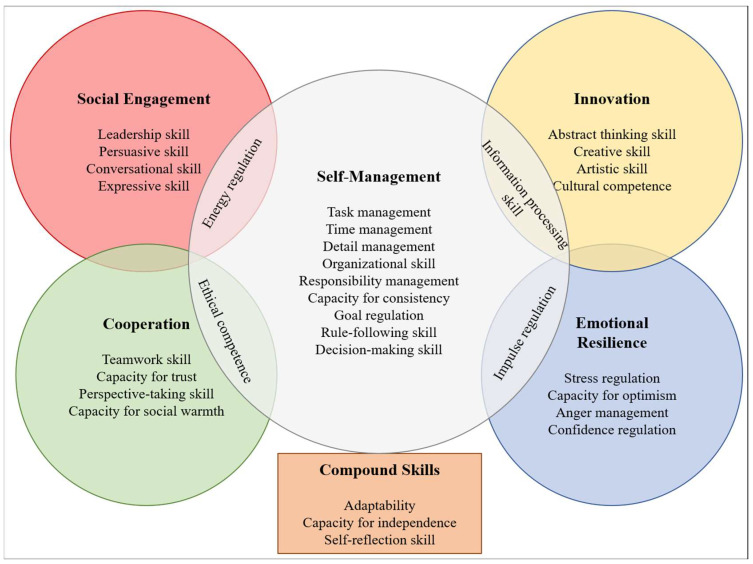
Skills included in the SEB framework and measured by the BESSI (image adapted from Soto et al. 2022a).

Three additional facets (i.e., adaptability, capacity for independence, and self-reflection skills) are treated as compound skills and do not belong to any specific domain because they are equally crucial for all the other domains. Figure 1 summarizes the skills and domains included in the BESSI framework. A definition of the 32 facets is not provided here, given our focus on the five domains, but it is available at http://www.sebskills.com/about-seb-skills.html (accessed on 12 March 2023).

#### 1.1.2. SEB Skills between 12 and 19 Years Old 

The years between ages 12 and 19 (mainly adolescence) are a period of significant changes for individuals. During this time, boys and girls face physical, behavioral, personality, social, and biological changes that test their well-being and success (Bramen et al. 2011; Costa et al. 2019; Dahl 2004; Hills and Byrne 2010; Miller and Halpern 2014; Soto et al. 2011; Yurgelun-Todd 2007). Indeed, adolescence is one of life’s most psychologically challenging periods and is associated with the most marked decline in the well-being of the entire life span (i.e., depression symptoms, low life satisfaction; Orben et al. 2022; Sawyer et al. 2018). To overcome these issues and successfully adapt through these transitions, Napolitano and colleagues (Napolitano et al. 2021) argue that adolescents will consistently use (and consequently develop) SEB skills, similarly to what happens to other important characteristics such as emotional intelligence, character strengths, prosocial behaviors, cognitive abilities, and emotion regulation (D’Amico and Geraci 2022; Demetriou et al. 2022b; Heintz and Ruch 2022; Ross et al. 2019). In particular, concerning SEB skills, Napolitano and colleagues (Napolitano et al. 2021) suppose that cognitive transitions (e.g., modification in brain structure, functionality, and connectivity) should lead to the development of perspective-taking (cooperation domain), abstract thinking (innovation domain), impulse regulation (at the border between emotional resilience and self-management domains), and goal regulation (self-management domain) skills, among the others. Additionally, SEB skills should gain importance because of the role they play in many social transitions that permeate adolescence (Napolitano et al. 2021): Indeed, between the end of primary school (i.e., around 10–11 years old) and the end of secondary school (i.e., around 17–19 years old), boys and girls are asked to make decisions about their future lives (e.g., course choices, job/university choices), gain responsibility, engage in extracurricular activities consistently, build their first intimate relationships, and engage with the larger community actively (e.g., civic responsibilities, participation in elections, volunteering). To efficiently manage all these requests and transitions to new roles and responsibilities, it is fundamental to have or develop higher self-management, cooperation, social engagement, innovation, and emotional resilience skills (Napolitano et al. 2021; Soto et al. 2022b). 

But do SEB skills scores change during this period? We will try to answer this question with a first cross-sectional analysis of the trajectories of mean-level scores in SEB skills between 12 and 19 years old.

#### 1.1.3. SEB Skills and Gender 

Despite SEB skills’ importance for both males and females, the challenges, variations, and situations they face are not the same, especially during adolescence (Kågesten et al. 2016). This might lead to differing development of SEB skills that could be highlighted by mean differences in SEB skills at different ages (i.e., males show higher/lower scores than females) but also by differences in the cross-sectional trajectories of SEB skills between 12 and 19 years old (i.e., the slope/trajectory of SEB skills varies between the two genders). In particular, gender differences might emerge from sources of variation, including genetic, biological, and hormonal differences, but also from the cultural and social environment they are exposed to. For example, an environment might frame women as more prone to social interactions than men, which can induce males and females to engage differently in social and cultural activities, such as sports, arts, or volunteering (Bramen et al. 2011; Eder and Parker 1987; Feraco and Meneghetti 2022; Gur et al. 2012; Ristori et al. 2020; Sawyer et al. 2018; Yurgelun-Todd 2007). In other words, if females engage less in team sports activities, they might develop less effective cooperation skills than males if participating in team sports increases cooperation skills. These suggestions are corroborated by findings that show that females outperform males in emotion recognition abilities, prosocial behaviors, empathy, and responsible decision-making (Allemand et al. 2015; Ross et al. 2019; Van der Graaff et al. 2014; Wright et al. 2018), but also from opposite findings showing that males outscore females in sport participation, emotional stability, self-efficacy, and activity levels (Deaner et al. 2012; Huang 2012; Moksnes et al. 2019; Soto 2016) during adolescence.

Based on these premises, do SEB skills scores and trajectories differ between males and females during adolescence?

### 1.2. Rationale of the Study

Soto et al. (2021, 2022a, 2022b) proposed the first integrative framework of clearly defined SEB skills, with initial empirical evidence showing that SEB skills matter for students and adolescents; they positively predict a host of positive scholastic and non-scholastic outcomes (Lechner et al. 2022; Sewell et al. 2023; Soto et al. 2022b), including academic achievement, life satisfaction, volunteering, and scholastic engagement. So far, however, no one has analyzed whether male and female adolescents differ in SEB skills and whether SEB skills scores vary between 12 and 19 years old, but theoretical foundations of the SEB framework and evidence from different constructs suggest that they should (Cabello et al. 2016; Demetriou et al. 2022b; Wright et al. 2018). Therefore, we cross-sectionally analyzed age and gender differences in SEB skills between 12 and 19 years old. An analysis of age × gender trends in SEB skills during this period of life is fundamental to uncover possible difficulties (e.g., a mismatch between the new skills requested and adolescents’ perceived abilities; steep declines in specific skills) that might be associated with new tasks, activities, or hormonal and personality variations that characterize males and females at varying degrees. Additionally, the same analysis could unveil the malleability of SEB skills between 12 and 19 years old, confirming Napolitano and colleagues’ (Napolitano et al. 2021) hypotheses and clarifying the skills that could be more or less affected by interventions at different ages.

### 1.3. Hypotheses

- Although this is the first study exploring age and gender differences in SEB skills, and we do not have specific hints from the literature about how they should vary during adolescence, we preregistered our hypotheses (https://osf.io/f5png) based on two large studies on personality traits (Soto 2016; Soto et al. 2011). On this basis, we expected that:
- Self-management skills should follow a U-shaped trajectory between 12 and 19 years old. This trajectory should be similar for males and females.
- Innovation skills are expected to be stable across ages and similar between males and females.
- Cooperation skills should be stable with age and higher in females than males.
- Social engagement skills should decrease with age and be higher in females than males.
- Emotional resilience skills should be higher in males than females, and such differences could increase through adolescence.

## 2. Materials and Methods

### 2.1. Participants

The sample was composed of 3112 participants enrolled by the authors for this study and 1218 participants obtained from two open datasets^1^ (542 participants, Lechner et al. 2022; 676 participants, Soto et al. 2022a) that collected data following a similar procedure and that included participants of the same age. After checking the data for careless responses, 221 participants were excluded, and the final sample included 4106 participants (2215 female; 54%) from 12 to 19 years old. These were well distributed across ages, except for 12-, 13-, and 19-year-old participants, who were less than 500 each (see Table 1 for the distribution of the participants for each age and gender). The study was approved by the University Ethics Committee for Research in Psychology.

### 2.2. Measures

Behavioral, Emotional, and Social Skills Inventory (BESSI; Soto et al. 2022a). It measures social, emotional, and behavioral skills and includes 192 items on a 5-point Likert scale. For each item, the participants grade their ability to perform the behavior, thought, or feeling described (from 1 = not at all well to 5 = exceptionally well). The BESSI-192 is designed to measure 32 facets (six items per facet), and the five overarching domains of self-management (e.g., “Plan out my time”), social engagement (e.g., “Lead a group of people”), cooperation (e.g., “Understand how other people feel”), emotional resilience (e.g., “Calm down when I’m feeling anxious”), and innovation skills (e.g., “Understand abstract ideas”). The measure showed excellent psychometric properties in the validation study (Cronbach’s alpha was higher than .80 in all samples and subscales). To achieve the aim of this study, we focused on the five domains of SEB skills, but supplementary analyses are also reported for the 32 facets. All the scores were calculated following Soto and colleagues’ (2022a) scoring method. The questionnaire and all the items are available at http://www.sebskills.com (accessed on 12 March 2023).

### 2.3. Procedure

To keep the data collection as representative as possible, hundreds of school directors and secretaries were contacted via email and invited to participate in the study. Thirty-three schools agreed to participate. The first author explained the study aims and procedures in detail to the schools responsible. Schools collected the consent forms from the parents of the students (or the students if 18 years old) and finally administered the questionnaire in class under teachers’ supervision. The questionnaire was administered in Qualtrics and took around 20 min to complete. The data collection started in November 2022 and ended in March 2023. The data collected in the two additional samples followed a similar procedure and were all collected online, even using different tools than Qualtrics (Lechner et al. 2022; Soto et al. 2022a). However, data from Lechner et al. (2022) were not collected in class.

### 2.4. Data Analysis

All the analyses were run using the R version 4.2.4 (R Core Team 2020) and were preregistered at https://osf.io/f5png (accessed on 24 February 2023). 

#### 2.4.1. Transformation and Inference Criteria

Given the large sample size, we did not rely on *p* values but always calculated effect sizes. Indeed, very small effects would be significant with this sample, even if they were practically negligible (i.e., a standardized difference of *d* = .06 and correlations of *r* = .03 would be significant). To make the results straightforward and comparable between dimensions, we scaled all the scores as *T*-scores (mean = 50, standard deviation = 10). Following Soto and colleagues (Soto 2016) and Cohen’s (1988) guidelines, we interpreted *T*-score differences as:

- Small if they amounted to ~2 *T*-points.
- Medium if they amounted to ~5 *T*-points.
- Large if they were higher than 8 *T*-points.

#### 2.4.2. Preliminary Analyses

First, *t*-tests were run to test the difference between males and females in the five SEB skill domains. Differences in *T*-scores (Δ*_T_*) and Cohen’s *d*s were calculated, with positive values indicating higher male scores. The correlation between SEB skills and participants’ age was also calculated separately for the total sample and males and females.

#### 2.4.3. Model Comparison

Given that the effect of age on psychological variables might not be linear and could differ between male and female adolescents, we adopted a model comparison approach to compare nested models that test for the linearity or non-linearity of the effects and their interaction with gender (as previously done in other studies; e.g., Ross et al. 2019). In particular, for each of the five dimensions, we ran:

- A null model (intercept only, m0) was used as a baseline for all comparisons.
- A model with only age as a linear predictor (m1) that only assumed an effect of age on the dependent variable.
- A model with interactions between age and gender (m2) assumed that the effect of age is linear but different between males and females.
- A model (m3) in which the same assumptions of m2 were made, but the effect of age was expected to follow a quadratic trend (i.e., the age trends follow a curvilinear pattern).
- A last model (m4) in which the effect of age was expected to follow a cubic curve (i.e., the curve goes up and down multiple times).

All the models were compared using the AIC index, and the model with the lowest AIC was selected as the best model (Wagenmakers and Farrell 2004). When the AIC value was the same between the two models, the most conservative model was selected. Results and statistical significance of the effects will be descriptively reported for each selected model, but because of the multiple effects and interactions that make the results difficult to interpret, we plotted all the data for a graphical interpretation (McElreath 2016).

#### 2.4.4. Graphical Analysis

To make the results easily understandable, data were plotted using loess smoothing with span = 1 to account for non-linear variations of SEB skills with age. Male and female scores in each dimension were plotted against age to inspect the interaction between the two terms graphically. To inspect the robustness of the findings, we also adopted a bootstrap procedure and plotted the results obtained from 150 random samples of 1000 participants sampled with replacement from the total sample.

#### 2.4.5. Additional Analysis

**Figure A1:**
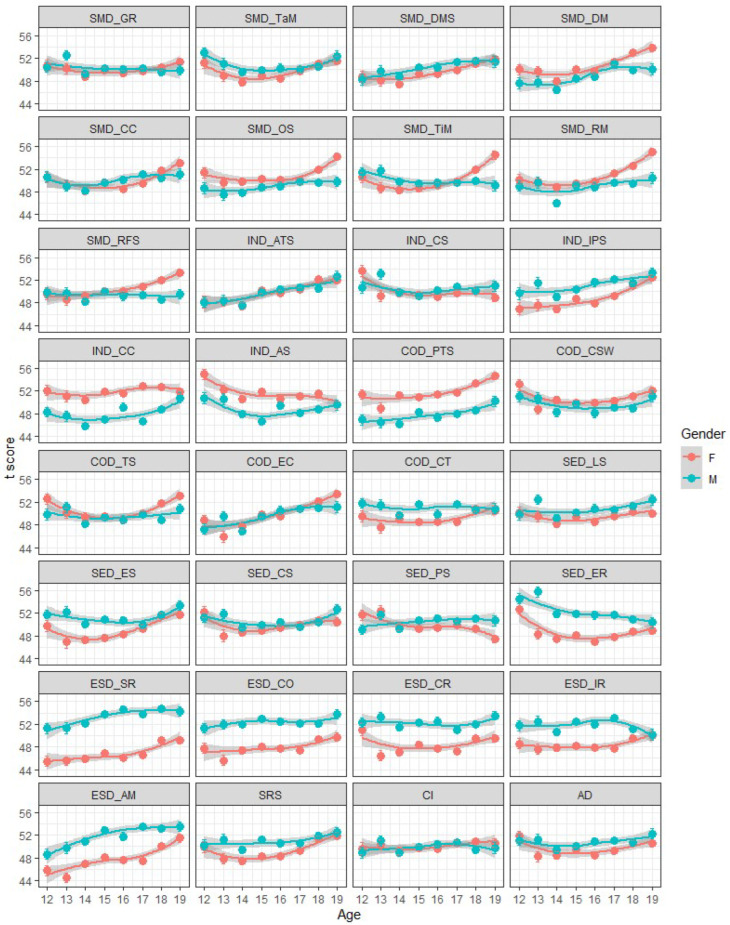
Trajectories of mean-level scores in the 32 SEB skills for males and females. *Note*. SMD = self-management domain; IND = innovation domain; COD = cooperation domain; SED = social engagement domain; ESD = emotional resilience domain; GR = goal regulation; TaM = task management; DMS = decision-making skills; DM = detail management; CC = capacity for consistency; OS = organizational skills; TiM = time management; RM = responsibility management; RFS = rule following skills; ATS = abstract thinking skills; CS = creative skills; IPS = information processing skills; CC = cultural competence; AS = artistic skills; PTS = perspective taking skills; CWS = capacity for social warmth; TS = teamwork skills; EC = ethical competence; CT = capacity for trust; LS = leadership skills; CS = conversational skills; PS = persuasive skills; ER = energy regulation; SR = stress regulation; CO = capacity for optimism; CR = confidence regulation; IR = impulse regulation; AM = anger management; SRS = self-reflection skills; CI = capacity for independence; AD = adaptability.

Graphical analysis was additionally applied to the 32 SEB facets to explore their trajectories. These results are only reported in Appendix A (Figure A1). 

## 3. Results

### 3.1. Preliminary Analysis

**Figure 2:**
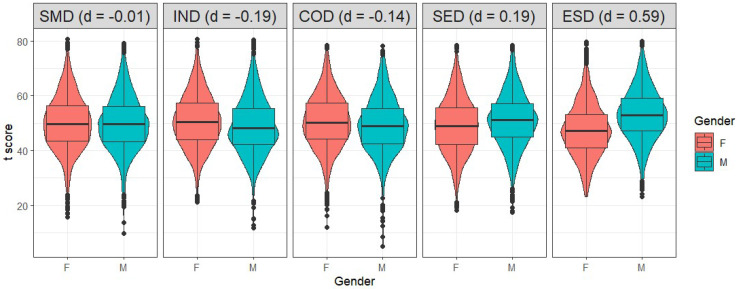
*T*-scores in the five BESSI domains for males and females. Cohen’s *d*s is reported in parentheses. *Note*. SMD = self-management domain; IND = innovation domain; COD = cooperation domain; SED = social engagement domain; ESD = emotional resilience domain.

Results of the correlation analysis showed that self-management, emotional resilience and cooperation skills increase with age in females (*r* = .14, .11, and .10, respectively). In contrast, linear correlations were mostly negligible in males (.02 < *r* < .07) and in the total sample (.02 < *r* .09). Concerning *t*-tests, mostly small or negligible gender differences emerged in the total sample (see Figure 2): males showed higher scores in social engagement (*d* = .19, Δ*_T_* = 1.86, *p* < .001) and emotional resilience (*d* = .59, Δ*_T_* = 5.66, *p* < .001) skills, but lower scores in innovation (*d* = −.19, Δ*_T_* = −1.88, *p* < .001) and cooperation skills (*d* = −.14, Δ*_T_* = −1.43, *p* < .001). No significant differences emerged for self-management skills (*d* = −.01, Δ*_T_* = −.12, *p* = .69). Given their magnitudes, the only noteworthy difference in the total sample regarded emotional resilience skills. All the other differences were mostly negligible.

### 3.2. Model Comparison and Graphical Analysis

**Figure 3:**
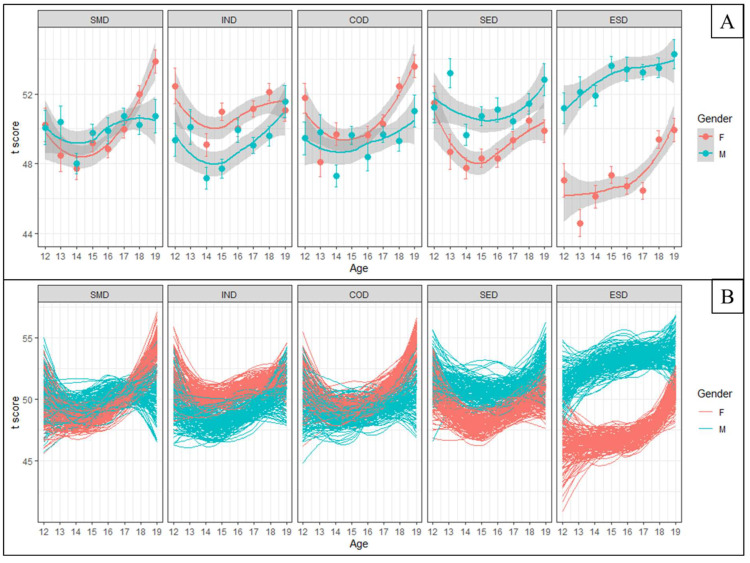
Graphical representation of SEB skill trajectories in male and female adolescents. (**Panel A**): Dots and bars represent mean scores and their standard deviations. The lines represent the predicted values of the smoothed model and their 95% confidence intervals in grey. (**Panel B**): lines represent the predicted values of every bootstrapped smoothed model. *Note*. SMD = self-management domain; IND = innovation domain; COD = cooperation domain; SED = social engagement domain; ESD = emotional resilience domain.

The model comparison procedure results are reported in Table 2 for each SEB domain. For all domains, the third (quadratic) or the fourth (cubic) model was the best (i.e., AIC was the lowest). Age trends are plotted in Figure 3.

- For self-management skills, the quadratic model with interaction was the best (m3). All effects within the model were also significant, highlighting a different curvilinear relationship between age and self-management skills in males and females.
- For innovation skills, the cubic model was selected (m4), but no significant interaction effects emerged from the results of the model. In other words, similar trajectories emerged in males and females.
- For cooperation skills, the quadratic model was selected (m3), but the interaction effect did not reach significance (*p* = .09), highlighting similar trajectories between males and females.
- For social engagement skills, the cubic model (m4) was selected. All effects within the model were also significant, highlighting a difference in the mean level and the trajectories between males and females.
- For emotional resilience skills, the quadratic model was selected (m3). Significant effects emerged for age and gender differences and their interaction, highlighting a general difference in the level of emotional resilience skills that, however, changes based on the different trajectories of males and females.

*T*-score differences between males and females are reported in Table 3 for each age and mainly show small or negligible differences. To make the results more understandable, all the data were plotted in the original form and with bootstrapping (see Figure 3).

### 3.3. Additional Analysis

The age trends of the 32 skills plotted in Figure A1 in Appendix A show variability between skills of the same domain: Within the same domain, some skills follow different trajectories, skills in which males score higher than females, and others in which the opposite happens (e.g., task management and detail management skills). However, a systematic analysis of these differences goes beyond the scope of our study and requires larger datasets. 

## 4. Discussion

Life, between 12 and 19 years of age, is characterized by big changes and adaptations: During this period, individuals face social, biological, cognitive, hormonal, and school changes and must adapt efficiently to all of them to maintain high levels of well-being and succeed at school or in other domains of their life. SEB skills are necessary (Feraco et al. 2023a; Napolitano et al. 2021; Ross et al. 2019; Steinberg 2008). Consequently, SEB skills are supposed to change and grow, but our results also show that—possibly caused by a marked increase in specific demands and responsibilities—adolescents’ self-perception of SEB skills also diminishes at some points, with differences emerging within and between genders.

### 4.1. SEB Skills Trajectories

Despite preliminary analysis showing negligible differences between males and females in all SEB domains except emotional resilience, and only small correlations between SEB skills and age in females, the situation appears more complicated and interesting when looking at SEB trajectories. Indeed, the best model was never linear; age always had a quadratic or even a cubic association with SEB skills. Additionally, such patterns differed between males and females in self-management, social engagement, and emotional resilience skills. 

#### 4.1.1. Self-Management Skills 

Between 12 and 19 years old, self-management skills increased by roughly 1 point (with ups and downs) in males and up to 5 points in females. Interestingly, self-management skills were more stable in males, while in females, they slightly decreased before 15 years old and then rose steeply, leading females to report higher scores than males at the end of adolescence (see Figure 3). This might be a cumulative effect of females’ increase in all SEB skills after 15 years old, which might help them manage emotions, relationships, and thoughts and consequently facilitate general self-management skills. Even if the results for self-management in males were not in line with our hypothesis of a U-shaped trajectory of self-management skills (Soto 2016; Soto et al. 2011), as suggested by Napolitano and colleagues (Napolitano et al. 2021), after primary school, children and adolescents are increasingly asked to become more independent, be responsible for their commitments, and regulate their behavior toward their current and future goals. This should be when self-management skills are acquired and used consistently and increasingly. Indeed, self-regulatory abilities, impulse control, goal settings, and decision-making abilities emerge during adolescence (Napolitano et al. 2011; Ross et al. 2019; Salmela-Aro 2009; Yurgelun-Todd 2007). The emergence of such skills is key for adolescents’ future success, but males probably need support to learn how to manage their tasks more efficiently and increase their self-management skills because, differently from females, this does not seem to happen naturally.

#### 4.1.2. Innovation Skills 

Concerning innovation skills, the trajectories followed a similar sinusoidal pattern in males and females, who also show small differences in their mean levels of innovation skills in favor of females. Mean values at 19 years old, however, were very similar. A first decline (albeit small) in innovation skills at the beginning of adolescence is in line with studies showing that traits such as curiosity, creativity, and openness generally decline at this age (Engel 2015; Heintz and Ruch 2022; Soto et al. 2011) and increase again in late adolescence. This is also in line with new developmental theories of personality and cognitive abilities that state that complex reasoning and abstract thinking skills acquire importance around 15–17 years old. In fact, at this age, boys and girls must face the new challenges and requests of the corresponding school period, in which they must consistently think about complex information and principles of topics such as physics, mathematics, and philosophy (Demetriou et al. 2022a, 2022b).

#### 4.1.3. Cooperation Skills 

Cooperation skills followed a curvilinear pattern with a negligible decrease before 15 years old and a steeper increase afterwards. Descriptively, females seem to have a steeper increase, but the interaction term in the model was not significant. Males’ scores increased slightly through adolescence (maximum Δ*_T_* ~ 3), while females showed a steep rise after 16 years old (maximum Δ*_T_* ~ 5). Mean levels appear slightly higher in females but only after 15 years old. These results do not align with our tentative hypotheses that predicted higher and more stable female scores through adolescence (Soto 2016). This differentiates skills from personality traits such as agreeableness. Indeed, while female adolescents tend to be more agreeable (Soto et al. 2011), males between 12 and 15 years old report being at least as good as their female counterparts in using those abilities linked with efficient cooperation with others, such as teamwork, trusting others, and understanding others’ positions and thoughts. In other words, we might speculate that while the tendency to be kind and agreeable remains stable or even decreases in adolescence (Soto 2016), adolescents’ ability to manage interpersonal relationships efficiently increases.

#### 4.1.4. Social Engagement Skills 

Females’ and males’ trajectories of social engagement between 12 and 19 years old show a U-shaped pattern. In particular, social engagement skills decline (steeply in females) between 12 and 15 years old (Δ*_T_* ~ 4) and increase after 15 years old (Δ*_T_* ~ 2). This is particularly interesting because social engagement skills are the only SEB domain that shows an apparent fall during the first years of adolescence in both genders. With our data, we can only observe the cross-sectional variation in SEB skills and cannot draw inferences on the causes of such variation, but we can speculate that the steep decline in social engagement skills is driven by the significant changes that individuals face in late childhood concerning their social relationships, which become central to their lives (Wentzel et al. 2010). In fact, they usually change schools and friends in this period and often develop their first intimate relationships. All these changes might test their social engagement abilities, and even if abilities per se do not decrease, they could feel or realize that their skills are no longer adequate to face the new social challenges and consequently report lower perceived abilities. Importantly, the decline seems limited to 12–15 years old and particularly concerns females. Later on, scores on social engagement skills increase. Contrary to our expectations, and differently from the Big Five, males show higher scores in social engagement skills compared to females. This suggests once again that skills and traits are distinguishable and that, in this case, females are generally more extroverted in adolescence. However, males still report a higher ability to behave successfully in social engagement situations.

#### 4.1.5. Emotional Resilience Skills

As expected, the biggest gender difference in emotional resilience skills concerning mean levels and trajectories emerged. Emotional resilience skills constantly increase in males (especially between 12 and 15 years old), while females show stable emotional resilience skills between 12 and 15 years old and then increase until 19 years old (see Figure 3). Importantly, the females’ increase is steep, and the difference with males decreases from almost 7 points (T scores) to 4 in late adolescence. In general, our finding is in line with research on neuroticism (Roberts and Yoon 2022; Soto 2016) but contradicts findings from other studies on emotional intelligence, suggesting that females outperform males in emotional intelligence tasks (Cabello et al. 2016; D’Amico and Geraci 2022). Males, however, tend to overestimate their abilities in self-reported emotional intelligence (D’Amico and Geraci 2022), which could also explain our result. What is important to note is that females show no increase in emotional resilience between 12 and 15 years old, suggesting that specific interventions might support adolescents’ social engagement, self-management, and emotional resilience skills during this period.

#### 4.1.6. A Brief Look at the 32 Skills 

Although an analysis of the 32 skills goes beyond the scope of this study, and results are not presented in depth, it seems evident that specific skills do not always follow the trajectories of their corresponding domain scores. For example, within the self-management domain, girls report higher scores in organizational skills and detail management and males in task management; additionally, females’ advantage in cooperation skills between 12 and 16 years old is probably driven by perspective-taking skills but not by the capacity for trust. Interestingly, gender differences always emerged between the five skills belonging to the emotional resilience domain. Future studies with larger samples might deepen these specific trajectories to provide more informative results about potential interventions.

#### 4.1.7. General Discussion 

In general, we found a complex pattern of developmental trends of SEB skills, and every domain showed peculiar age trajectories that (in some cases) differed as a function of gender. Interestingly, most of our findings were not in line or were only partially in line with previous research on personality (Soto et al. 2011). This may suggest that skills are not only important in predicting positive outcomes above and beyond personality traits but that they also follow different and specific patterns of development. Among the five domains, skills seem to increase during adolescence, but self-management, innovation, and social engagement skills decline between 12 and 16 years old. This may suggest that it is particularly important to focus on these skills before the end of primary school to help boys and girls face the future social and emotional challenges of adolescence and possibly maintain better well-being. Specifically, interventions or activities targeting social engagement and emotional resilience in female children and adolescents may be crucial, while males may benefit more from developing self-management, cooperation, and innovation skills. To achieve these goals, extracurricular activities such as volunteering, scouting, sports, and artistic activities can play a significant role (Feraco et al. 2022; Khasanzyanova 2017). These activities allow participants to explore new and challenging situations, expand their interpersonal relationships, acquire new skills, and put their abilities to the test (Eccles et al. 2003). Furthermore, schools and professionals can propose structured interventions that parallel social-emotional learning interventions (Taylor et al. 2017) or strength-based interventions (Lavy 2020; Niemiec 2017) and emotional intelligence training (D’Amico 2018). However, it is essential to note that the effectiveness of such interventions and activities on SEB skills, as measured by the BESSI, requires further testing, and our suggestions should be taken with caution. Finally, future studies focusing on the 32 facets may help identify narrower targets for interventions, making them more specific to individuals’ needs.

### 4.2. Limitations and Future Directions

Despite the large sample and the novelty and importance of the field (European Commission 2016; Soto et al. 2021), our work has limitations that must be addressed in future studies. First, our analysis is based on cross-sectional data, and all the developmental variations we found could therefore subsume a true change as well as cohort effects or sampling variability effects at different ages. Future studies should consider within-individual variability using longitudinal approaches to prevent cohort effects. Second, we did not control for different constructs, such as personality traits that may partially covary with skills, and we could not disentangle SEB skill development from personality changes; longitudinal cross-lagged designs could be helpful to achieve this aim. Similarly, we only highlighted age differences without providing data that might explain such changes (except for gender) that, for example, might be related to the practice of extracurricular activities, social support, and teachers’ and peers’ relationships (Feraco and Meneghetti 2022; Shiner and Caspi 2003; Van den Akker et al. 2014), but also to biological and genetic factors (Bramen et al. 2011; DeYoung et al. 2010; Sawyer et al. 2018). Future studies should test the drivers of SEB skills’ development.

## 5. Conclusions

Our study is the first to analyze cross-sectional trajectories of the development of SEB skills in male and female adolescents between 12 and 19 years of age. In line with expectations (Napolitano et al. 2021), our results strongly suggest that adolescence is a period of high malleability and variation of SEB skills. Additionally, each skill showed a specific pattern of development that often differed between males and females and from expected changes in personality. These findings might inform interventions and policies targeting the development of SEB skills to sustain adolescents’ well-being and success, but future studies should aim to understand better why SEB skills change and what we can do to facilitate their development or prevent their decline at various ages.

## Figures and Tables

**Table 1:** Distribution of male and female participants for each age bracket.

	12	13	14	15	16	17	18	19	Total
Males	130	118	265	350	212	404	274	138	1891
Females	113	112	252	367	376	397	381	217	2215
Total	243	230	517	717	588	801	655	355	4106

**Table 2:** AIC indices of all the models. The best models are in bold.

Model	SMD	IND	COD	SED	ESD
m0: intercept	30564	30564	30564	30564	30564
m1: + age	30528	30554	30541	30564	30546
m2: + age × gender	30523	30524	30523	30530	30191
m3: + age2	**30502**	30514	**30503**	30516	**30186**
m4: + age3	30503	**30511**	30507	**30512**	30189

*Note*. SMD = self-management domain; IND = innovation domain; COD = cooperation domain; SED = social engagement domain; ESD = emotional resilience domain.

**Table 3:** Differences between males and females at each age. Negative values indicate a difference in favor of females. Differences bigger than |2| are in bold.

	12	13	14	15	16	17	18	19
SMD	−0.14	1.93	0.30	0.55	1.06	0.76	−1.76	**−3.13**
IND	**−3.09**	0.02	−1.93	**−3.26**	−0.10	**−2.10**	**−2.50**	0.50
COD	**−2.30**	1.70	**−2.39**	−0.02	−1.27	−0.60	**−3.12**	**−2.56**
SED	−0.29	**4.52**	1.89	**2.44**	**2.78**	1.09	0.97	**2.93**
ESD	**4.15**	**7.52**	**5.78**	**6.28**	**6.71**	**6.82**	**4.11**	**4.33**

*Note*. SMD = self-management domain; IND = innovation domain; COD = cooperation domain; SED = social engagement domain; ESD = emotional resilience domain.

## Data Availability

The data presented in this study are available on request from the corresponding author. The data are not publicly available due to ongoing data collection. Data will be made freely available after the end of the longitudinal data collection.

## Note

1 To ensure that the results are valid independently from the sample, we performed a bootstrap analysis. No specific analysis was run on the individual datasets given the scarcity of participants at each age in the online samples (i.e., less than 50 female or male participants and no participants less than 14 years old), which makes it impossible to draw inferences on the mean scores of the population at that specific age.
